# Detection of diarrhoea associated rotavirus and co-infection with diarrhoeagenic pathogens in the Littoral region of Cameroon using ELISA, RT-PCR and Luminex xTAG GPP assays

**DOI:** 10.1186/s12879-021-06318-x

**Published:** 2021-06-28

**Authors:** Rahinatou N. Ghapoutsa, Maurice Boda, Rashi Gautam, Valantine Ngum Ndze, Akongnwi E. Mugyia, Francois-Xavier Etoa, Michael D. Bowen, Mathew D. Esona

**Affiliations:** 1 Department of Biochemistry, Faculty of Science, The University of Yaoundé 1, Yaoundé, Cameroon; 2 Department of Microbiology, Faculty of Science, The University of Yaoundé 1, Yaoundé, Cameroon; 3 Division of Viral Diseases, National Center for Immunization and Respiratory Diseases, Centers for Diseases Control and Prevention, Atlanta, Georgia USA; 4 Faculty of Health Sciences, University of Buea, Buea, Cameroon; 5 Department of Microbiology and Parasitology, Faculty of Science, University of Buea, Buea, Cameroon

**Keywords:** Cameroon, Rotavirus, Childhood diarrhoea, RT-PCR, Co-infection, Rotarix, xTAG GPP

## Abstract

**Background:**

Despite the global roll-out of rotavirus vaccines (RotaTeq/Rotarix / ROTAVAC/Rotasiil), mortality and morbidity due to group A rotavirus (RVA) remains high in sub-Saharan Africa, causing 104,000 deaths and 600,000 hospitalizations yearly. In Cameroon, Rotarix™ was introduced in March 2014, but, routine laboratory diagnosis of rotavirus infection is not yet a common practice, and vaccine effectiveness studies to determine the impact of vaccine introduction have not been done. Thus, studies examining RVA prevalence post vaccine introduction are needed. The study aim was to determine RVA prevalence in severe diarrhoea cases in Littoral region, Cameroon and investigate the role of other diarrheagenic pathogens in RVA-positive cases.

**Methods:**

We carried out a study among hospitalized children < 5 years of age, presenting with acute gastroenteritis in selected hospitals of the Littoral region of Cameroon, from May 2015 to April 2016. Diarrheic stool samples and socio-demographic data including immunization and breastfeeding status were collected from these participating children. Samples were screened by ELISA (ProSpecT™ Rotavirus) for detection of RVA antigen and by gel-based RT-PCR for detection of the VP6 gene. Co-infection was assessed by multiplexed molecular detection of diarrheal pathogens using the Luminex xTAG GPP assay.

**Results:**

The ELISA assay detected RVA antigen in 54.6% (71/130) of specimens, with 45, positive by VP6 RT-PCR and 54, positive using Luminex xTAG GPP. Luminex GPP was able to detect all 45 VP6 RT-PCR positive samples. Co-infections were found in 63.0% (34/54) of Luminex positive RVA infections, with Shigella (35.3%; 12/34) and ETEC (29.4%; 10/34) detected frequently. Of the 71 ELISA positive RVA cases, 57.8% (41/71) were fully vaccinated, receiving two doses of Rotarix.

**Conclusion:**

This study provides insight on RVA prevalence in Cameroon, which could be useful for post-vaccine epidemiological studies, highlights higher than expected RVA prevalence in vaccinated children hospitalized for diarrhoea and provides the trend of RVA co-infection with other enteric pathogens. RVA genotyping is needed to determine circulating rotavirus genotypes in Cameroon, including those causing disease in vaccinated children.

## Background

Diarrhea is the second most common cause of childhood mortality worldwide, with Group A Rotavirus (RVA) recognized as a leading diarrhoeagenic agent, causing 38% of acute diarrhea among children aged five and below worldwide [1, 2]. RVA remains a major cause of diarrhea related deaths among children and infants aged < 5 in Sub-Sahara Africa, accounting for 104,000 deaths and 600,000 hospitalizations yearly [3, 4]. The practice of good hygiene helps to limit the transmission of all diarrhoeal pathogens, vaccination remains the most effective method of RVA prevention. The roll-out of four oral vaccines, RotaTeq, Rotarix®, ROTAVAC, and Rotasiil, approved and recommended by WHO, has significantly reduced worldwide mortality and morbidity associated with RVA [5]. In the USA and in Canada, the number of RVA-related hospitalizations has decreased significantly, to about 70,000, with no more than 60 deaths per year [6, 7]. Compared to high income countries, alleviating RVA burden in sub-Saharan Africa with an efficacious vaccine is progressing slowly [6, 7]. The poor performance of vaccine may be explained in part by malnutrition, which has a negative impact on immunogenic development [3, 8], or the high level of RVA exposure associated with unhealthy hygienic conditions and contaminated drinking water supplies, or by the possibility that the vaccine used may not effectively protect against circulating strains [3, 8].

Since 2009 in low-income countries, the number of RVA related deaths and morbidity has dropped following the introduction of the RVA vaccines [9–11]. From 2015 to 2016, the prevalence of diarrhea due to RVA decreased from 38.3 to 12.2% in Mozambique and from 50.8 to 29% in Swaziland from 2013 to 2016. In South Africa, the hospitalization rate dropped by one third during the same period [12, 13]. In Togo, the introduction of Rotarix in June 2014 has already shown remarkable results over the past 2 years, with a 53% decrease in RVA related hospitalization [14].

On March 28th, 2014, Cameroon officially introduced Rotarix into its Expanded Program on Immunization (EPI) [15]. During the pre-vaccination period, RVA caused more than 5800 deaths yearly among children under 5 years of age in Cameroon and accounted for 33 to 38.1% of diarrhea-related hospitalizations in this age group [15, 16]. In addition, previous studies in Cameroon showed the presence of rare genotypes and regional genotypic diversity [17–21]. The circulating strains in many regions of Cameroon including the Littoral, the East and South regions were still unknown both prior to and after vaccine introduction. The aim of this study was to establish an epidemiology update, useful for RVA infection follow up, for evolution and trend study, as well for the initiation of a vaccine impact study. It describes the frequency of RVA infection in the Littoral region of Cameroon among children aged five and under, hospitalized for severe diarrhea, in relation to their RVA vaccination status, co-infection with other enteric pathogens, as well as their feeding mode during the first 6 months of life.

## Methods

### Ethical and administrative considerations

Prior to conducting this study, ethical clearance (N°2016/01/696/CNERSH/SP) and research authorization were obtained from the Littoral regional delegation of Public Health (N°: 1684/AR/MINSANTE/DRSPL/BCASS). A signed informed consent was obtained from the parent or the legal guardian of all the patients sampled. The study protocol conformed to the ethical guidelines of the 1975 declaration of Helsinki was approved by the National Ethics Committee and the Ministry of Public Health of Cameroon.

### Study population, period and sample collection

From May 2015 to April 2016, we carried out a cross-sectional study among hospitalized children under 5 years of age, presenting with acute gastroenteritis in the following hospitals in the Littoral region of Cameroon; the Douala Laquintinie hospital, the district hospitals of Bonassama and Deido, and the regional hospitals of Edéa and Nkongsamba. A diarrheic stool sample was collected from each participating hospitalized child along with socio-demographic information including RVA immunization and breastfeeding status which were obtained from the patient medical record and/or from guardians. The stool specimens were transported in a cooler (4 °C to 8 °C) following recommended standard to the virology laboratory of the Mother and Child Centre of the Chantal Biya’s Foundation, Yaoundé, and stored in aliquots of 1.5 mL at − 80 °C until analyzed. Prior to storage, a 10% (v/v) suspension to be used for total RNA extraction was prepared for each sample using nuclease-free distilled water (Invitrogen™) as diluent, then stored at − 20 °C until used.

### RVA antigen screening by ELISA

RVA detection was done using the ProSpecT™ Rotavirus ELISA kit (Thermo Scientific, Oxoid, UK) following the manufacturer’s protocol. All samples and reagents were brought to laboratory temperature before use. A 10% (v/v) dilution of each sample was prepared using the kit provided specimen dilution buffer. The dilution buffer served as a negative control (NC), while known laboratory positive and negative samples served respectively, as additional positive and negative controls.

Supernatants from test samples and controls (100 μL) were transferred into respective wells of the precoated microtiter plate, and the subsequent reaction steps were performed as per protocol [22]. The reading of the results after addition of TMB substrate was first done by visually observing the color change in the wells containing the positive samples and comparing with the NC well. Confirmation of the results was done by spectrophotometric reading at 450 nm after the reaction was stopped with a solution of 0.46 M sulfuric acid. Positive results were confirmed if optical density (OD) value was greater than or equal to negative control OD + 0.2 [22]. Only ELISA positive samples were selected for further downstream tests.

### Molecular detection of the VP6 gene by RT-PCR

#### Extraction of total viral RNA

RNA extraction of 10% stool suspensions was manually performed using the QIAamp RNA mini spin kit (Qiagen, Hilden, Germany) following the manufacturer’s instructions. The eluted RNA extract was stored at − 80 °C until used.

#### One step RT-PCR

The extracted RNA was subjected to VP6 gene detection by gel-based RT-PC [18, 23], (Qiagen, Inc.,Valencia, CA, USA) using the primers:

VP6F (nt 747–766) 5′ GACGGVGCRACTACATGGT 3′ and 21.

74VP6R (nt 1126 to 1106) 5′ GTCCAATTCATNCCTGGTGG 3′ [24].

RNA sample (4 μL) was mixed with 3 μl of primer mix (20 μM) in 0.5 ml PCR tube/well, vortexed and centrifuged at 8000 rpm for 10 s, then denatured at 97 °C for 4 min and rapidly cooled for 1 min on ice. The mixture was centrifuged briefly then placed back on ice, and 23 μL of master mix (made of H_2_O, 16 μL; Qiagen One step RT-PCR buffer 5X, 5 μL; dNTP (10 mM), 1 μL; Qiagen One step RT-PCR enzyme, 1 μL) was added in the tube. The reaction was amplified using an ABI 9700 thermocycler under the following conditions: 42 °C, 30 min; 95 °C, 15 min; and 30 cycles of 94 °C 30s, 42 °C 30s, 72 °C 45 s; with a final cycle of 7 min at 72 °C, then 4 °C on hold [18, 23]. The amplification products were separated on a 2% agarose gel (Invitrogen) containing 10 μL of red gel (Biotium), with a 100 bp marker (Invitrogen), and the bands were visualized using a “Gel Doc TMXR +” illuminator (BIO-RAD).

#### Luminex xTAG GPP assay

Luminex xTAG GPP testing was performed in the Rotavirus Surveillance and Molecular Epidemiology Team Laboratory at the Centers for Disease Control and Prevention (CDC), Atlanta, USA. Total nucleic acid was extracted from 10% suspension (in nuclease free water) of stool samples using the MagMAX™ Total Nucleic Acid Isolation Kit (Ambion Life Technologies) as described previously [25]. MS2 bacteriophage served as internal process control for the extraction process and the purified total nucleic acid extract was tested with the xTAG GPP on MAgPix instrument (Luminex Corporation, Austin, TX) following the manufacturer’s instructions for multiplex assay. The targeted gastrointestinal pathogens included Cryptosporidium spp., *Entamoeba histolytica*, *Giardia lamblia*, *Campylobacter* spp., *Clostridium difficile* toxin A and B, Enterotoxigenic *Escherichia coli* (ETEC) heat-labile (LT) and heat-stable (ST) toxins, *Escherichia coli* O157, Shiga toxin producing *Escherichia coli* (STEC), *Salmonella* spp., *Shigella* spp., *Vibrio cholerae*, *Yersinia enterocolitica*, Adenovirus 40/41, Norovirus and RVA (https://www.luminexcorp.com/gastrointestinal-pathogen-panel/).

### Statistical analysis

All statistical analysis was performed using SPSS v26.0 software to look for associations between vaccination or breastfeeding and RVA infection. Statistical distributions were compared using the chi-square test, for categorical variables with a confidence interval of 95% and statistical significance was considered at a *p* ≤ 0.05.

## Results

From May 2015 to April 2016, diarrheic stool samples were collected from 130 children of both sexes, hospitalized for severe diarrhea in the Littoral region of Cameroon. Seventy-nine, representing 60.8% of these children had received the two doses of Rotarix vaccine, 20% (26/130) were not vaccinated, and 19.2% (25/130) had unknown vaccination status. The ELISA assay detected rotavirus antigen in 54.6% (71/130) of diarrheic stool specimens. There was no statistically significant difference in rotavirus detection among vaccinated (57.8% (41/71) RVA positive versus 64.4% (38/59) RVA negative), non-vaccinated (21.1% (15/71) RVA positive versus 18.6% (11/59) RVA negative) and unknown vaccination status (21.1% (15/71) RVA positive versus 17.0% (10/59) RVA negative; *p* = 0.786) groups.

**Fig. 1:**
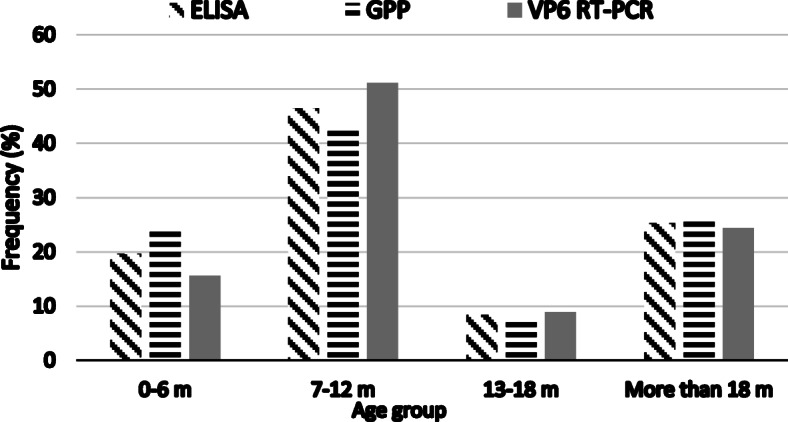
Group A rotavirus distribution by age group based on the different diagnostic methods performed

All age groups were affected, but children aged 7–12 months were the most affected with 46,5% (33/71) positive cases (Fig. 1). No statistically significant difference was observed in RVA detection among the breastfed (45.1%, 32/71) RVA positive versus 44.1% (26/59) RVA negative) and mixed feeding (54.9% (39/71) RVA positive versus 55.9% (33/59) RVA negative) groups (*p* = 0.76).

ELISA positive samples (*n* = 71) were further subjected to VP6 gene detection by RT-PCR for confirmation. To assess co-infection, we chose the Luminex xTAG GPP assay, which also served as a second confirmation assay for RVA. A summarized in Tables 1, 63.4% (45/71) of cases were RVA positive by RT-PCR and 76.1% (54/71) were RVA positive using the xTAG GPP assay (Table 1).

**Table 1:** General features of the laboratory results

Test results			Number of cases
ELISA+	GPP+	PCR+	45
ELISA+	GPP+	PCR-	9
ELISA+	GPP-	PCR-	17
**Total**			71

**Key: +: positive; −: negative;**

The results of xTAG GPP assay revealed that 57.0% (31/54) were from Rotarix vaccinated children. The statistical analysis indicated no link between the feeding mode and RVA positivity determined by Luminex xTAG GPP (*p* = 0.864).

The summary of the laboratory results shows that 45 samples were positive by all 3 methods, ELISA, GPP, RT-PCR. Seventeen ELISA positive samples tested negative for RVA with xTAG GPP assay, and 9 ELISA positive samples were RVA positive with xTAG GPP assay and negative VP6 gene by RT-PCR (Table 1).

The number of RVA co-infection with other gastroenteric pathogens was high 63.0% (34/54). We identified 13 co-infection patterns, 8 cases with more than two pathogens (Table 2). *Shigella* was the most frequent coinfecting pathogen 35.3% (12/34), followed by ETEC with 29.4% (10/34). Other co-infecting pathogens included, Adenovirus (20.6%), *Campylobacter* spp. (14.7%), Norovirus GI/GI (11.8%), *Cryptosporidium* (5.8%) and *Salmonella* spp. (5.9%). The GPP test showed 10 cases of infections or co-infections not involving rotavirus and 7 negative samples.

**Table 2:** Co-infections patterns and the number of occurrences

S/N	Co-infection patterns	Occurrences
1	RVA, Shigella	9
2	RVA, ETEC LT/ST	3
3	RVA, Adenovirus 40/41	6
4	RVA, Campylobacter	3
5	RVA, Norovirus GI/GII	2
6	RVA, Salmonella 1, Salmonella 2	2
7	RVA, Cryptosporidium	1
8	RVA, Campylobacter, ETEC LT/ST	2
9	RVA, Adenovirus 40/41, ETEC LT/ST	1
10	RVA, ETEC LT/ST, Shigella	2
11	RVA, Norovirus GI/GII, Shigella	1
12	RVA, ETEC LT/ST, Cryptosporidium	1
13	RVA, Norovirus GI/GII, ETEC LT/ST	1

## Discussion

When Cameroon introduced Rotarix into the EPI [15], no epidemiological data and information on the circulating strains of RVA in the Littoral, the South and the East regions of Cameroon were available. The present study carried out 1 year after introduction of RVA vaccine in Cameroon is the first investigation on RVA infection in the Littoral region. This study provides an update of rotavirus epidemiology in Cameroon and highlights the importance of co-infection with other enteric diarrhoeagenic pathogens in RVA positive cases. Using antigen detection by ELISA, 54.6% of the sampled children tested positive for RVA. This frequency is higher than the global proportion of diarrheal cases attributed to rotavirus, and also higher than the 42.8 and 41.0% RVA prevalence previously obtained in Cameroon in 2012 [23] and in 2014 [21]. A concern is that the previous lower prevalence results by Ndze and his team as well as Boula and collaborators represent RVA prevalence before the introduction of the rotavirus vaccine [21, 23]. Although RVA prevalence during the pre-vaccine and the post-vaccine periods may have remained the same, the absolute number of rotavirus cases and admissions may have declined, and additional data will have to be collected to investigate this finding. The higher ELISA prevalence reported in this study may also be attributed to false positive ELISA results. Ye et al., (2013) observed a discrepancy between ELISA and RT-PCR in Australia, and false positive ELISA reactions were identified as the cause. They noted that twenty-five samples positive by ELISA were negative for VP6 detection by gel-based RT-PCR [26]. The observed discrepancy might as well result from low viral load in samples, due to additional shipment and freeze-thaw cycles, yet, with enough VP6 protein present to be detected by ELISA. Discrepancies of similar order between ELISA and VP6 RT-PCR were also observed recently by McAuliffe et al. (2018, 2019) in New-Zealand [27, 28].

The GPP results confirmed that 76.1% (54/71) of RVA positive samples detected using ELISA contained RVA. GPP is an automated multiplex nucleic detection acid test with a high sensitivity (95.8%) and specificity (100%) for RVA targets [25]. The GPP is considered the most reliable and sensitive method for RVA screening [29]. However, many African countries use ELISA to screen for RVA and evaluate the progress and impact of the rotavirus vaccine [11, 13, 14]. Further investigations are needed to confirm the impact of ELISA false positive in epidemiological studies.

High prevalence of RVA infection in Cameroon (> 41%) may be partly explained by a number of socio-environmental conditions, including difficult access to potable water, and the poor economic situation of the country, which negatively impact the standard of living and the sanitary conditions, compounded by poor hygiene and malnutrition. In effect, there is a gradient in RVA vaccine effectiveness between high, medium and low-income countries. Vaccine effectiveness is generally better in high income countries and poor in low-income countries [4, 5]. Malnutrition, a poverty related conditions of low-income countries, has been identified as a contributing factor for reduced vaccine effectiveness and has been experimentally proven to impair the adaptive immune response to RVA infection in gnotobiotic piglets [30]. Therefore, malnutrition may lead to impaired development of protective immune responses following vaccination and increase the susceptibility of the host to RVA infection. In addition, poor sanitary conditions and lack of potable water increase the exposure to other enteric pathogens, a condition known to inhibit immune responses to RVA vaccine [31].

In this study, we found that 63.0% of RVA cases detected were also infected with other enteric pathogens. RVA cases co-infected with other enteric pathogens (*Entamoeba histolytica*, *Giardia lamblia*, Plesiomonas shigelloides, *Escherichia coli*, Salmonella spp., Shigella spp., Norovirus and Adenovirus) are common among children age less than 5 years in low-income countries [32, 33]. Furthermore, RVA infection predisposes enterocytes to increased adhesion and invasion by other enteric pathogens and the presence of coinfecting pathogens increases RVA replication in the host gut [34]. The co-infection frequency of RVA cases with other enteric pathogens found in this study is about six times (10.3%) that observed in Niger by Langendorf and collaborators in 2015 [35]. Co-infection with Shigella, ETEC, Adenovirus and Campylobacter are likely to increase the severity of the diarrhea and may cause an increase in diarrheal related deaths among children aged less than 5 years, thus increasing the diarrheal burden [36]. Moreover, the pathogenic processes of co-infecting pathogens can act synergistically to worsen the patient conditions, resulting in a greater burden of the infection [32]. RVA infections decrease nutrient absorption through depletion of enterocytes but does not induce intestinal inflammation. *Shigella* causes inflammatory and invasive diarrhea, while ETEC alters intestinal epithelial integrity [33, 37]. These combined effects may trigger several cellular mechanisms that can exacerbate diarrheal disease.

The main limitations of this study were reduced access to medical records, which increased the number of children with unknown vaccination status, a large number of non-consenting mothers or legal guardians affected enrolment and inability to differentiate between circulating RVA and RVA vaccine strains. Due to limited resources, we could test only ELISA positive samples by GPP and PCR; assay comparison would have been more robust if all the samples were tested by the three methods.

## Conclusions

This study revealed a high prevalence of RVA among vaccinated children admitted to hospital for the treatment of diarrhea, with significant presence of co-infections with other diarrheagenic pathogens. RVA genotyping is needed to determine which genotypes are circulating in the Littoral region of Cameroon, including those causing disease in vaccinated children. This study, which demonstrates a high percentage of RVA co-infection with other pathogens, suggests the need to carry out expanded studies on RVA co-infections in Cameroon in order to provide appropriate measures for management and control of infectious diarrheal diseases among children below 5 years of age. It is also imperative to assess the impact of Rotarix in the socio-environmental context of Cameroon to identify key factors impairing successful development of adequate protection against RVA.

## Data Availability

The datasets used and analyzed during the current study are available from the corresponding author.

## Abbreviations

CDC: Center for disease control and prevention
dNTP: Desoxyribonucleotides triphosphate
ELISA: Enzyme linked immunosorbent assay
EPE: Expanded program on immunization
ETEC: Enterotoxigenic *Escherichia coli*
GPP: Gastrointestinal pathogen panel
NTC: Non-template control
OD: Optical density
RNA: Ribonucleic acid
RT-PCR: Reverse transcriptase-polymerase chain reaction
RVA: Group a rotavirus
STEC: Shiga toxin producing *Escherichia coli*
TMB: Tetramethylbenzidine
WHO: World health organization
